# Applying machine learning to safe vascular anastomosis

**DOI:** 10.1016/j.jpra.2025.06.008

**Published:** 2025-06-13

**Authors:** Hiroki Umezawa, Akatsuki Kondo, Marie Taga, Rei Ogawa

**Affiliations:** Department of Plastic, Reconstructive and Aesthetic Surgery, Nippon Medical School Hospital, 1-1-5 Sendagi, Bunkyo-ku, Tokyo, Japan

**Keywords:** Microsurgery, Vascular anastomosis, Artificial intelligence, Machine-learning, Real-time processing

## Abstract

**Background:**

Machine-learning technology is currently being introduced into the medical field and has been shown to aid diagnostic imaging, patient examinations, patient-data analysis, various surgical aspects, and medical education. Recent advances in exoscopes and monitors are prompting a shift from optical microscope-based microsurgery to heads-up microsurgery. The high-definition exoscope images are highly suitable for machine learning. Since an algorithm that detects predictive signs of thrombus formation would aid microsurgery and help train surgeons to identify vessels at risk of unsafe microvascular anastomosis, we here asked whether we could use exoscope images to train such a machine-learning algorithm.

**Methods:**

Arterial clots, intimal-wall damage, debris, and stumps in 9150 ORBEYE™ exoscope images of arterial anastomosis obtained in 2023–2024 were annotated with RectLabel pro™. These images were used to train the You Only Look Once (YOLO) model (Ultralytics) to detect the thrombus-predicting signs. The YOLO code was executed within Google Colaboratory™.

**Results:**

After algorithm training for 100 epochs, the four objects were detected in real time, albeit with high levels of false-positive and false-negative detections.

**Conclusion:**

Our study shows the potential of machine learning on exoscope images to generate algorithms that promote safe microsurgical anastomosis. It also shows how the recent emergence of Python code, Google Colaboratory™, and machine-learning models such as YOLO has made it possible for even programming amateurs to develop effective machine-learning algorithms. Further development of new central and graphics processing units and computational processing methods will likely lead to machine-learning applications that improve surgery and facilitate medical training.

## Introduction

Machine learning is a branch of artificial intelligence where statistical algorithms learn patterns or relationships from data and generalize that learning to unseen data, thus performing tasks without explicit programming. It functions like neural networks. Typically, it involves input artificial neurons/nodes that send data/stimuli to a so-called hidden layer of neurons/nodes, which initially provide random weights to these inputs and sum them to produce an output that is detected by output neurons. The size of the hidden-layer outputs determines the function of the output neurons. If the output-neuron functions provide an erroneous outcome relative to the original input data, it is quantified as ‘loss function’. The error is then backpropagated through the neural network, thereby inducing the hidden neurons/nodes to adjust the weights that are attributed to the input stimuli. This backpropagation is repeated until the most accurate output is obtained. Machine-learning research started in the 1950s and was then propelled forward by the development of decision trees and cascade classifiers in the 1970s, the Viola-Jones detector and support-vector machines in the 2000s, and deep learning after 2010. The latter has led to machine-learning methods that use feedforward neural networks, convolutional neural networks (CNNs), and transformers, either alone or in various combinations.^1^, ^2^, ^3^, ^4^, ^5^, ^6^, ^7^, ^8^, ^9^

Machine learning has been shown to be extremely useful in the medical field, including surgery. For example, it can predict the likelihood of surgical complications and mortality, thus permitting surgeons to identify high-risk patients and apply interventions that improve outcomes. It can also be used to identify and then detect diagnostic markers, or critical anatomical landmarks that help predict the optimal surgical route. Moreover, machine learning can aid surgical training by analyzing videos of trainees performing a procedure and providing feedback. The latter tasks are mediated by the great capacity of machine learning to detect objects. This capacity was bolstered recently by developments that caused object-detection algorithms to shift from those that rely on methods such as sliding windows and region proposals (which consume significant computational resources and are thus unsuitable for real-time processing)^1^, ^2^, ^3^ to algorithms such as YOLO (You Only Look Once).^6^ The CNN-based architecture of YOLO uses a single neural network that divides each image into a grid, with each grid cell predicting bounding boxes and class probabilities. This approach significantly accelerates computational processing, making it capable of real-time processing while maintaining accurate object detection.^6^^,^^8^ The recent emergence of publically accessible tools such as Python, Google Colaboratory™, Google Gemini™, and ChatGPT™ has also greatly simplified and democratized the development of machine-learning algorithms, thus allowing people without extensive programming backgrounds to develop and utilize this technology.

Microsurgery is highly useful for replanting amputated fingers, suturing nerves, and transferring free flaps and is thus frequently used to manage traumatic injuries and other conditions.^16^^,^^17^ Microsurgery was completely dependent on optical microscopes until recently, when exoscopes arose in conjunction with high-definition 4 K monitors.^10^, ^11^, ^12^ This technology has multiple benefits: the heads-up approach reduces the physical burden on surgeons, it helps secure of the surgical field of view, it facilitates indocyanine-green testing, it helps educate surgeons, and it simplifies the sharing of information between the surgical team members. Looking through the optical microscope can put strain on the neck and back because the body is fixed. By wearing very light 3D goggles and performing head-up surgery, the surgery can be completed in a comfortable position. If it is only vascular anastomosis, it takes 20–30 min, but if the surgeon lacks experience, if some trouble occurs, or if the surgery involves multiple blood vessels and nerves anastomosis, it becomes necessary to look through the microscope for a long time, which can cause a more noticeable strain.^10^, ^11^, ^12^ Significantly, exoscopes also provide very high-quality high-definition images that are suitable for analysis with machine-learning algorithms. However, there is currently relatively little research on machine-learning assistance in microsurgery, particularly with the YOLO algorithm.

There are many factors that can affect flap transplant surgery, including flap elevation, the condition of the recipient, flap placement, and thrombus formation etc. One of the key predictors of microsurgical failure is the formation of thrombus after vascular anastomosis. This itself is predicted by blood clots inside the blood vessels, damage to the vascular intima, and debris around the vessels just before the vessels are anastomosis.^13^, ^14^, ^15^, ^16^ Since these physical changes can be seen on exoscope images and thus theoretically recognized by machine learning, we here asked whether exoscope images could be used to train a machine-learning algorithm to detect in real time microvessels that are at risk of thrombus formation after microsurgical vascular anastomosis.

## Materials and methods

We adhered to the Strengthening the Reporting of Observational Studies in Epidemiology (STROBE) guidelines throughout the study design, data collection, and reporting process, ensuring rigor and transparency in our observational research methodology. And this retrospective single-center study was conducted in a tertiary-referral hospital (Nippon Medical School Hospital, Tokyo, Japan). It adhered to the principles of the Declaration of Helsinki and was approved by the institutional review board of Nippon Medical School Hospital (Approval No B-2024–934). All patients consented in writing to the possibility that their surgery-related data would be used for research purposes.

The exoscope-imaging data that were acquired during exoscope microsurgery procedures that were conducted at our hospital between June 2023 and April 2024 served as the supervised machine-learning image data. The exoscope was ORBEYE™ (Olympus) and its high-definition 3-dimensional images were projected on a 55-inch 4 K high-resolution monitor (LMD-XH550ST; Sony Olympus Medical Solutions Inc.). Specifically, 9150 still images from recorded exoscope movies showing arterial blood vessels just before vascular anastomosis were collected. Of these, 7000 were used for training and 2150 for validation. RectLabel pro™ was used to annotate the following four objects in the training images: (1) vascular stumps, (2) thrombi, (3) damage to the vascular wall, and (4) rubbish (i.e., materials floating in or around the blood vessel). The machine-learning process was conducted with a graphics processing unit (GPU) from Google Colaboratory™, which is a collaborative platform that facilitates computations with cloud services. This GPU was used together with the YOLO version 8 (YOLOv8) object-detection model (Ultralytics) to construct the trained algorithm. YOLOv8 is an open-source model that was developed by Ultralytics. The source code and model weights are available under the MIT license. The original code can be found at https://github.com/ultralytics/yolov8.

A real-time object-detection application was developed by using Visual Studio Code™. For this, a weight file that was downloaded from a training model created in Google Colaboratory™ was leveraged. The entire implementation was conducted in Python code. We also used ChatGPT™ to correct and refine Python code issues. We thought that by using these tools for development, including programming, we could significantly reduce costs.

## Results

### Clinical and educational benefits

Since important tissues and areas requiring attention can be visually highlighted in real time, surgeons, assistants, residents, and medical students were able to share the same information. Real-time display also enabled a more detailed understanding of the process by which thrombogenic factors, which affect the outcome of vascular anastomosis, were removed. Regarding the educational benefits, this approach was particularly effective in explaining techniques and helping residents and medical students understand them, as it allowed them to focus on important points highlighted in real time. In some cases, surgeons were able to identify and remove thrombogenic factors that might have been overlooked due to fatigue or other factors.

### Training of machine learning

**Figure 1:**
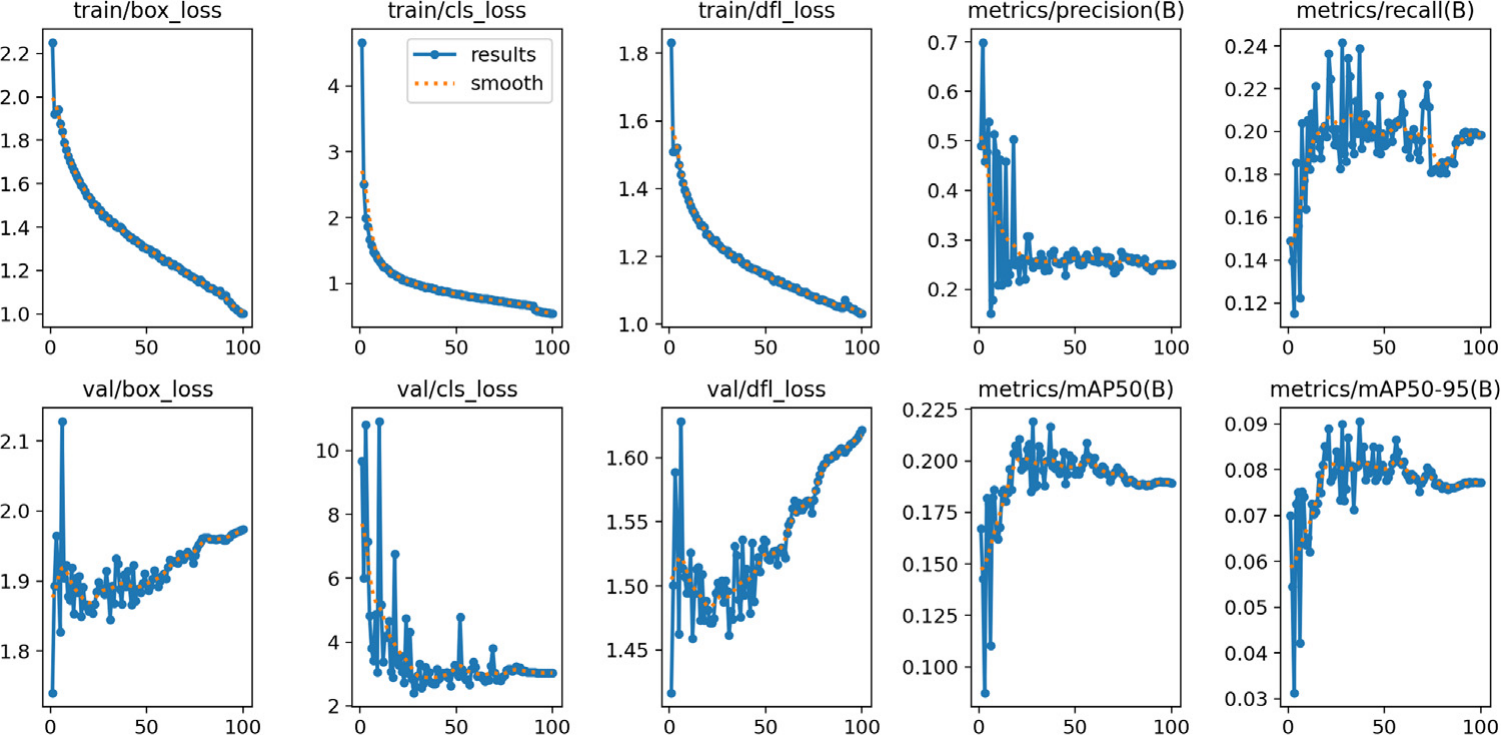
Effect of epoch number on loss function measures, precision, and recall. (Top) Seven thousand exoscope images of arterial microvessels just before anastomosis were annotated for the presence of vessel stumps, clots, intimal-wall damage, and rubbish. The annotated images were then used to train our YOLOv8 algorithm. (Bottom) A separate set of 2150 exoscope images was used to validate the trained algorithm. train, training dataset; box_loss, box loss; cls_loss, class loss; dfl_loss, distribution focal loss; precision(B), best precision; recall(B), best recall; val, validation dataset; mAP50, mean average precision (mAP) at an intersection over union (IoU) of 0.50; mAP50–95, average of the mAP at IoU thresholds ranging from 0.50 to 0.95.

We first trained our YOLOv8 algorithm on 7,000 exoscope images of arterial microvessels that were about to be anastomosed by microsurgery. Thus, the entire training dataset was passed through the algorithm repeatedly (each pass is termed an epoch) and the error of the output relative to input was quantified after each pass as loss function. Three different loss functions were calculated: (i) Box Loss (box_loss), which measures the accuracy with which the algorithm places a 2-dimensional bounding box on a specific object in the image (e.g., vessel clots); (ii) Class Loss (cls_loss), which measures the accuracy with which the algorithm classifies the different objects in the image (e.g., clots and vessel-wall damage); and (iii) Distribution Focal Loss (dfl_loss), which measures the accuracy with which the algorithm discerns between very similar objects in the image on the basis of their fine features and spatial information. We found that as the number of epochs approached 100, the three loss functions decreased from 2.2, 4.7, and 1.8, respectively to 1.0, 0.5, and 1.1, respectively: the latter values are considered to be adequate loss values (Figure 1, top). Thus, the algorithm learned after 100 epochs to accurately detect the objects in the exoscope images, thereby reducing the discrepancy between the predicted and actual values.

**Figure 2:**
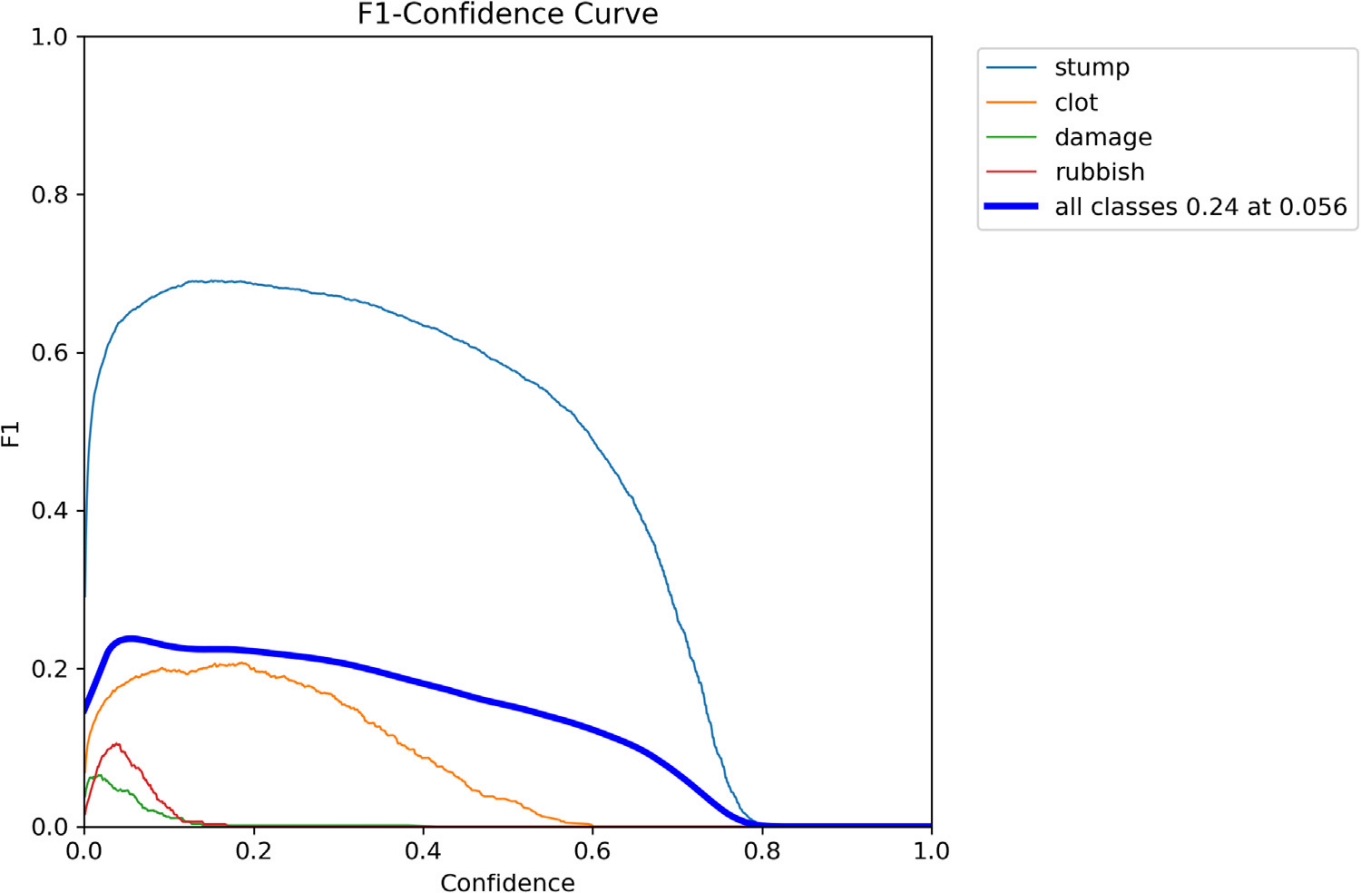
F1 curve showing the precision and recall of the learning model.

Two other important measures of object detection by machine-learning algorithms are (i) Best Precision (precision(B)), which measures the frequency with which the algorithm correctly detects the objects in a target class; and (ii) Best Recall (recall(B)), which measures the frequency with the algorithm detects all objects in each target class. High precision and recall values indicate low rates of false positives and false negatives, respectively. Both precision(B) and recall(B) report the best value that was achieved during the training epoch. In our study, both precision metrics changed over 100 epochs from 0.7 to 0.12, respectively to 0.26 and 0.20, respectively (Figure 1, top). Both of these performances are poor. The F1 curve was then assessed. This provides the weighted harmonic mean (F1 score) of the precision and recall of each object class: thus, both metrics have the same importance. The confidence values that optimized the precision and recall for stump, clot, vessel damage, and rubbish were 0.2, 0.2, 0.001, and 0.05, respectively and corresponded to maximum F1 values of 0.7, 0.2, 0.08, and 0.1, respectively. For the whole model with all four classes, the optimal confidence value was 0.056, which corresponded to the maximal F1 value of 0.24 (Figure 2). Thus, while the model detects many of the objects, it also has many false-positive and false-negative detections.

**Figure 3:**
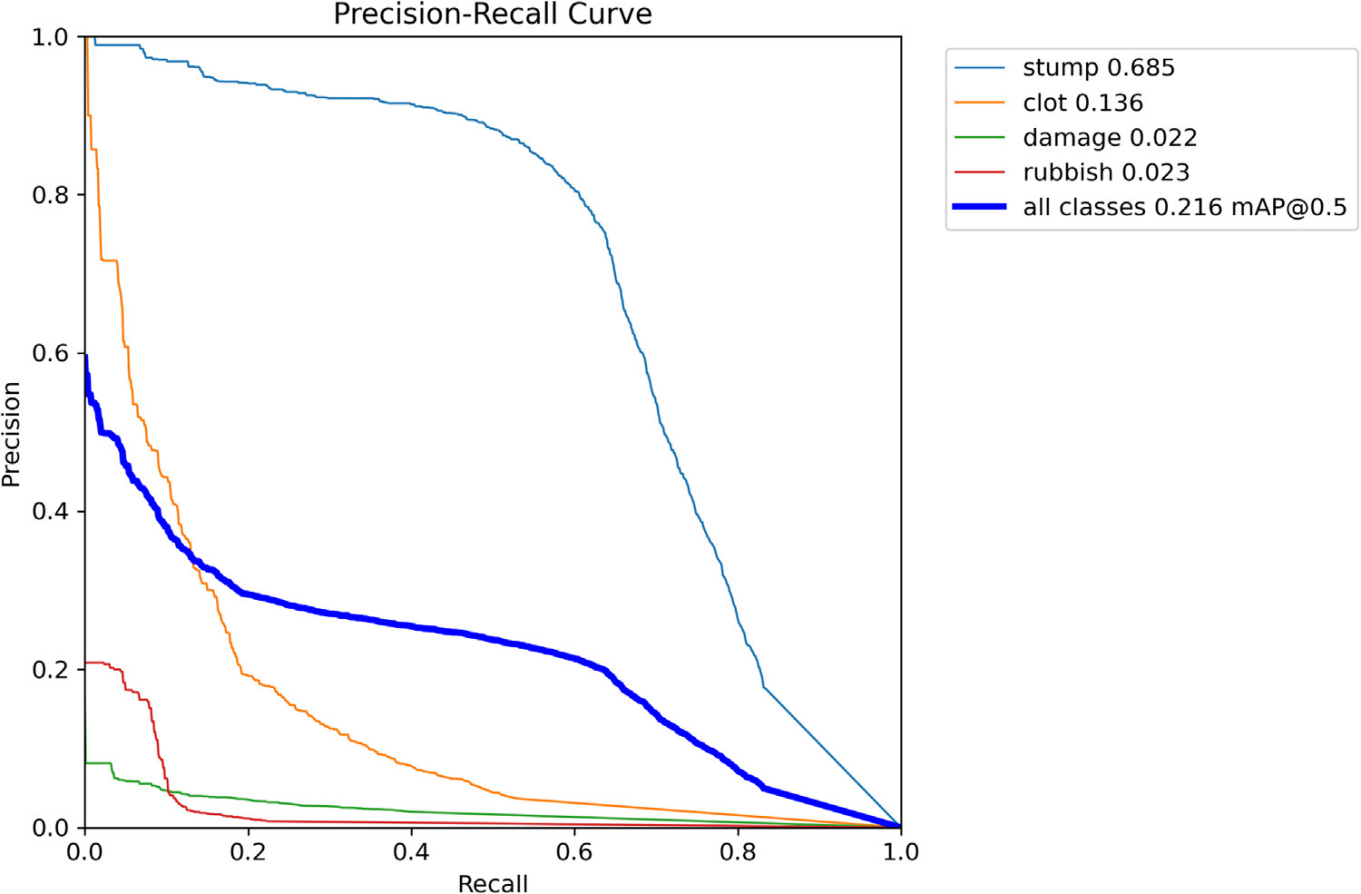
Precision-recall curve showing the relationship between precision and recall in the learning model.

To assess the relationship between precision and recall more closely, we generated the Precision-Recall Curve. This illustrates the trade-off between precision and recall at different confidence thresholds. The closer the curve comes to the upper-right corner, the better the performance of the model. Our Precision-Recall Curve showed that while vessel stumps were detected with good precision, the other object categories were detected poorly (Figure 3). This is also observed when looking at the boundary boxes for the four object classes on still images: while the vessel stump, vessel damage and rubbish were detected accurately (Video.1).

Video 2 displays a video of microsurgical anastomosis under an exoscope that shows the algorithm detected vessel stumps, blood clots, and damaged vessel walls in real time. However, false positive and negative detections were also observed.

### Validation of machine learning

The trained algorithm was then run on the 2,150 exoscope images of microvessels that were about to be anastomosed by microsurgery. The Box Loss, Class Loss, and Distribution Focal Loss measures of function loss showed that the algorithm eventually stably classified the four object categories with moderate accuracy (cls_loss = 3.0 after 100 epochs). However, it could not stably place the bounding boxes on the four object categories accurately (box_loss = 2.0 after 100 epochs). It also could not stably differentiate very similar objects on the basis of their fine features and spatial information (dfl_loss = 1.6 after 100 epochs) (Figure 1, bottom). We also measured the precision with which the algorithm detected the target objects by calculating mAP50 and mAP50–95. mAP50 measures the mean Average Precision (mAP; the area under the Precision-recall Curve for all object classes) at an Intersection over Union (IoU) of 0.50. IoU quantifies the overlap between the predicted bounding box and the ground-truth bounding box. Thus, mAP50 measures how accurately the algorithm makes the “easy” detections. mAP50–95 is the average of the mAP at IoU thresholds ranging from 0.50 to 0.95. It provides a comprehensive overview of the performance of the algorithm across different levels of detection difficulty. In our study, mAP50 and mAP50–95 stabilized over 100 epochs during validation but both did so at low levels (0.195 and 0.08 at 100 epochs, respectively) (Figure 1, bottom).

Currently, false positives are detected and displayed, but there is a discrepancy between the calculated false positive rate and the bounding box of the false positive that continues to be displayed. In most cases, false positives appear for just a moment, like a bug, and then disappear immediately. The images are displayed on a sub-monitor separate from the monitor used for surgery, and an experienced doctor judges whether they are false positives or not.

The development cost was $30 for using Rectrabel pro and $20 for two months using Goocle colaboratory, totaling $70 including expenses.

## Discussion

A variety of factors are required for successful vascular anastomosis surgery. As Dr Acland explains, there are many points that need to be considered, such as how to use tools, gentle maneuvers, handling of tissues around the blood vessels, protecting the vascular intima, and cleaning the inside of the blood vessels.^16^ The system described in this report focuses on factors of thrombus formation. This is because it simplifies the system and increases the calculation speed within the computer, and it makes it easier to take images in the same surgical field and introduce machine learning.

By introducing this system, it is believed that it will be possible to reduce surgeons' oversights and minimize vascular anastomosis errors. During actual surgery, even experienced surgeons may occasionally fail to perform vascular anastomosis correctly due to factors such as fatigue or the assistance of inexperienced personnel. Even if the probability of error is low, it is a common goal for surgeons to reduce the failure rate as close to 0 % as possible. Furthermore, the more complex the surgery, the fewer the number of experienced instructors available. If this system can provide the same level of guidance as an experienced surgeon, it is expected to enhance the overall quality of surgery.

This system also proved effective in educating residents and medical students. Since it can identify thrombogenic factors in real time, it was possible to highlight key points and allow observers to learn how to handle such situations appropriately. This is considered highly effective because it allows explanatory videos to be shown without disturbing the surgeon and assistants who are focused on the procedure.

In the present study, high-definition images that were obtained with the ORBEYE™ exoscope just before microsurgical anastomosis were used to train an algorithm to detect signs that predicted thrombus, namely, clots and intimal-wall damage in the microvessels and debris around the microvessels. We found that while our trained algorithm did have high rates of false-positive and false-negative detection, it could detect all three signs in real-time. Thus, further work that focuses on reducing false positives and false negatives could yield an algorithm that effectively detects hallmark signs of microsurgical failure. Such an algorithm has the potential to help secure the surgical field of view and train surgical staff to detect microvessels that are at risk of thrombus formation. This study thus demonstrates the potential of real-time object-detection machine-learning technology to improve patient outcomes in various surgical or diagnostic settings.

In terms of our algorithm development process, we chose YOLOv8 over DETR (detection transformers),^9^ which is another promising method that is based on a transformer architecture consisting of a CNN encoder and a transformer decoder. In DETR, the input image is converted into a feature map by the CNN, after which the transformer decoder predicts the position and class of objects. However, although this method may lead to more accurate detection, the calculation costs are high.^6^^,^^8^

We also chose YOLOv8 because the class loss and object loss are more directly presented than in previous YOLO versions. This makes the loss function more concise without losing any information.^18^, ^19^, ^20^

We used Rectlabel Pro™ to annotate the four object classes (vessel stumps, clots, intimal-wall damage, and debris). It accounted for almost 90 % of the work time in this study and required a great deal of patience. It is recommended to undertake such work by a team with unified intent.

The annotated files were then analyzed with YOLOv8 in the Google Colaboratory™ environment to create a weight file. We found that the best results were obtained with 100 epochs and when the image size was set to 1,024 pixels. Increasing the epoch number or image size significantly extended the processing time. Conversely, when the epoch number was reduced to 50 or the image size to 640 pixels to improve processing speed, the detection capability of the algorithm decreased. The development of new central processing units, GPUs, and computational processing methods in the future may help to improve detailed detection and process more frames, thus reducing the false-positive and false-negative detections of our algorithm without requiring excessive processing time.

We used the weight file obtained with 100 epochs and 1,024-pixel image size to create an object-detection application on a local computer. Depending on the performance of the computer, this could potentially slow down image analysis. Nonetheless, our study showed that the four object classes could be detected in real-time and that it is possible to readily create applications that are sufficiently durable for clinical use.

To improve the accuracy of this system, it is necessary to continue fundamental updates in the future. A total of 9150 images were used for annotation in this study, but we believe that it is necessary to accumulate up to 40,000 images to further improve the accuracy of analysis. Additionally, by continuing research and increasing the number of annotated classes from the current four to six or eight, and combining them with sentence generation AI, it may be possible to provide even more accurate sentence-based suggestions. Therefore, we believe that continuing research across multiple facilities is essential.

The current limitations of this study include its implementation at a single institution, which limits the ability to reflect the opinions of a diverse range of surgeons. For instance, real-time object analysis is performed through computer processing, but image frames must be dropped during this process. This means that the number of images displayed per second is reduced compared to normal circumstances, and the optimal number of frames required to perform accurate analysis without causing noticeable delays during surgery has not yet been determined. However, since there are no specific restrictions on the types of images used for recognition training, it is believed that more accurate training data can be provided if more data is collected from more facilities and with more equipment. Further research is urgently needed.

## Conclusions

Our study supports the notion that machine learning could help improve real-time decision-making in surgery as well as aid other medical fields such as diagnostics. It also shows that the emergence of publically accessible tools such as Python, Google Colaboratory™, Google Gemini™, and ChatGPT™ enables even those without extensive programming backgrounds to develop machine-learning applications. Thus, these initiatives have democratized innovation in medical technology. Future research on integrating algorithms such as ours with robotic surgical systems or creating new algorithms for use in other surgical procedures is likely to improve patient outcomes and operational efficiency.

## Ethical approval

This retrospective single-center study was conducted in a tertiary-referral hospital (Nippon Medical School Hospital, Tokyo, Japan). It adhered to the principles of the Declaration of Helsinki and was approved by the institutional review board of Nippon Medical School Hospital (Approval No B-2024–934).

## Funding

The authors have no financial disclosures to make.

## Declaration of competing interest

The authors have no conflicts of interest to declare.

## Appendix Supplementary materials

Video. 1. This video demonstrated the object detection in recorded vascular anastomosis video. Detection by the trained algorithm of blood-vessel stumps and damage and rubbish around the blood vessels in an exoscope image. Image, video 1

Video 2. Video showing real-time object detection by our trained algorithm of factors that affect the success of vascular anastomosis. Image, video 2
